# MRI-assessed diaphragmatic function can predict frequent acute exacerbation of COPD: a prospective observational study based on telehealth-based monitoring system

**DOI:** 10.1186/s12890-022-02254-x

**Published:** 2022-11-23

**Authors:** Shuoshuo Wei, Rong Lu, Zhengping Zhang, Faxuan Wang, Hai Tan, Xiaohong Wang, Jinlan Ma, Yating Zhang, Ning Deng, Juan Chen

**Affiliations:** 1 Department of Pulmonary and Critical Care Medicine, General Hospital of Ningxia Medical University, Yongan Lane, Xingqing District, Yinchuan, 750004 Ningxia China; 2 Ningxia Medical University, Yinchuan, 750004 Ningxia China; 3 Department of Radiology, General Hospital of Ningxia Medical University, Yinchuan, 750004 Ningxia China; 4 Department of Critical Care Medicine, General Hospital of Ningxia Medical University, Yinchuan, 750004 Ningxia China; 5 School of Public Health and Management, Ningxia Medical University, Yinchuan, China; 6 Ministry of Education Key Laboratory of Biomedical Engineering, College of Biomedical Engineering and Instrument Science, Zhejiang University, 38 Zheda Road, Hangzhou, 310027 Zhejiang China; 7 Department of Pulmonary Medicine, People’s Hospital of Wuzhong, Wuzhong, 751100 Ningxia China

**Keywords:** Acute exacerbation, Chronic obstructive pulmonary disease, Closed-loop care pathway, Diaphragm function, Magnetic resonance imaging

## Abstract

**Background:**

Acute exacerbations of chronic obstructive pulmonary disease (AECOPD) have considerably high mortality and re-hospitalisation rate. Diaphragmatic dysfunction (DD) is common in COPD patients. However, whether diaphragmatic dysfunction is related to acute exacerbation is yet to be elucidated. This study aimed to evaluate the diaphragm function by magnetic resonance imaging (MRI) in COPD patients and assess whether the impact of DD may help predict AECOPD.

**Methods:**

20 healthy adult volunteers and 80 COPD patients were enrolled. The diaphragms function parameters were accessed by MRI. Patients were guided to start self-management by the Telehealth-based monitoring system following the enrolment. Events of acute exacerbation of COPD were recorded by the system and confirmed by healthcare providers. Binary univariate and multivariate logistic regression analyses were performed to investigate the factors associated with the frequency of AECOPD. Receiver operating characteristic (ROC) curves were further used to assess the value of prediction indexes.

**Results:**

Fifty-nine COPD patients completed a one-year follow-up based on the Telehealth-based monitoring system. The clinical outcomes showed that the diaphragm function parameters at the end of maximal breathing were lower in the COPD group than in the healthy control group (*P* < 0.05). ANOVA showed significant differences among Global Initiative for Chronic Obstructive Lung Disease (GOLD) stages for diaphragm function parameters, including chest wall motion, lung area, upper-lower diameter, and the diaphragm thickening fraction at the end of maximal breathing (*P* < 0.05). Moreover, significant differences in diaphragm function parameters were observed between patients with infrequent AECOPD (n = 28) and frequent AECOPD (n = 31) based on the frequency of AECOPD (*P* < 0.05). The diaphragm thickening fraction and the chest wall motion were associated with AECOPD after adjusting for age, sex, BMI, and lung functions, and the combination of predictions showed better accuracy in predicting the frequency of AECOPD.

**Conclusions:**

In COPD patients, diaphragm function parameters correlate with the severity of airflow limitation. The diaphragm thickening fraction and the chest wall motion were associated with the frequency of AECOPD and can predict it.

**Supplementary Information:**

The online version contains supplementary material available at 10.1186/s12890-022-02254-x.

## Background

Chronic Obstructive Pulmonary Disease (COPD) is characterised by persistent respiratory symptoms and airflow limitation. Its worldwide prevalence is increasing, and it is estimated that COPD will be the fourth leading cause of death by 2030 [1]. Acute exacerbation of COPD (AECOPD) plays a central role in the natural history of the disease, and its effect should not be underestimated. An exacerbation of COPD is defined as an acute worsening of respiratory symptoms that results in additional therapy [2]. AECOPD are complicate complications usually associated with increased airway inflammation, mucus production, and gas trapping, which contribute to increased dyspnoea, cough, and wheeze. Some patients are particularly susceptible to exacerbations and show worse health status, considerably decreased pulmonary function, exacerbated disease progression than those who have infrequent exacerbations, leading to severe morbidity and mortality [3]_。_ The COPD phenotype reflects the differences between individuals with COPD [4]. The exacerbation rates vary significantly among patients and during follow-up [5, 6]. Clinically, patients with frequent acute exacerbation have a higher hospitalisation rate than those with infrequent exacerbation. In addition, the acuteness of disease progresses further increases the difficulty in treatment of this disease [7]. The predictor of frequent exacerbations (defined as two or more exacerbations per year) is a history of previously treated events [5], and deteriorating airflow limitation is associated with an increasing prevalence of AECOPD [8]. Moreover, skeletal muscle, including diaphragm weakness, is associated with an increased risk for more severe exacerbations and increased hospital re-admission rate after an exacerbation [9].

The diaphragm is the most important respiratory muscle and plays a significant role in maintaining ventilation [10]. Diaphragmatic dysfunction occurs in patients with COPD, and changes in diaphragm structure are closely related to diaphragmatic dysfunction (DD) [11–14]. Patients with COPD have difficulty inhaling as the diaphragm, the agonist muscle of respiration, becomes short and flat [15]. This reduced respiratory function can be compensated by the increased activities of the respiratory synergistic muscles. Previous studies revealed the relationship between DD and high mortality, poor effect of treatments, and prolonged hospital stay in patients with AECOPD. During severe AECOPD, progressive development of dynamic hyperinflation causes a change in the geometry of the chest wall and diaphragm, with impaired lung function [16, 17]. Another study confirmed that lower diaphragm thickening fraction (DTF_max_) and diaphragm excursion (DE_max_) during maximal deep breathing were associated with AECOPD, which leads to the possibility of an imaging biomarker distinguishing AECOPD from stable status [18]. The impairment of diaphragmatic function may be one of the main pathophysiological mechanisms in COPD that seems related to acute exacerbation [19]^.^ However, little is known about the role of the diaphragm on COPD exacerbation, and whether DD is a predictor of AECOPD needs to be further investigated.

COPD exacerbations are usually identified based on an increase in various symptoms, including increased breathlessness and sputum production [20]. It has been known that the objective markers for exacerbations such as the use of antibiotics and steroids and whether the patient was admitted to the hospital or attended accident and emergency [1].However, it is estimated that less than a third of deteriorations are reported. Unreported exacerbations may not be serious enough to warrant an emergency visit or hospitalization [21]. Consequently, patient education on when to seek medical attention for exacerbations is of vital importance. In the management of COPD, some researchers have argued that remote monitoring may be a promising alternative or an adjunct to traditional health care services [22]. In recent years, many studies have established risk factors prediction models for acute exacerbation in COPD patients through different monitoring methods, including electronic medical records [23], questionnaire diary records [24], or telephone follow-up [25]. Nonetheless, it is not always possible to monitor all exacerbations in terms of frequency, severity, and likely causes. Due to these factors, acute exacerbations of COPD patients are underestimated, which renders that a dynamically monitoring COPD symptoms is crucial. In a recently published study, we designed and validated a feasible Telehealth-based monitoring system to monitor COPD acute exacerbations [26]. Under this system, acute exacerbation events were detected and further confirmed by a health care provider. Therefore, in this study, we hypothesize that diaphragmatic dysfunction in COPD patients and the diaphragm function worsens in frequent AECOPD patients compared with infrequent AECOPD patients, DD thus can help predict patients likely with frequency of AECOPD.

## Materials and methods

### Study design and data collection

This prospective observational study was conducted at the General Hospital of Ningxia Medical University from September 2017 to December 2019 and approved by the Ethics Committee for the Conduct of Human Research at the General Hospital of Ningxia Medical University (No.2017–206). All participants provided written informed consent after being informed of the possible risks of the study.

### Study population

The patients in the COPD group were recruited from outpatient clinics of the General Hospital of Ningxia Medical University. The inclusion criteria of enrolment included (1) age ≥ 40 years; (2) confirmed diagnosis of COPD according to the Global Initiative for Chronic Obstructive Lung Disease (GOLD) guideline [1]; (3) stable phase of COPD, no visit to the outpatient or emergency department, and no hospitalisation for AECOPD during the past 12 weeks; (4) access to a smartphone. The exclusion criteria were any of the following: the presence of pulmonary diseases besides COPD, such as pleural effusion, pneumothorax, phrenic nerve palsy, and interstitial lung disease; neuromuscular disease or chest wall deformities; metallic materials in the body; claustrophobia; patients with mental disorders; and participation in other studies.

#### Healthy volunteers (healthy control group)

A total of 20 healthy volunteers were recruited from the local community, all of whom were non-smokers and whose pulmonary function tests were normal. The healthy control group was selected based on the following criteria: (1) age was older than 20 without any evidence or history of pulmonary disease; (2) no noticeable emphysematous changes on chest radiographs; (3) no previous thoracic surgery; (4) voluntary participation.

The exclusion criteria were any of the following: (1) individuals with a history of thoracic/upper abdominal surgery; (2) known diaphragmatic paralysis; (3) chronic neuromuscular disease; (4) significant underlying respiratory diseases; (5) claustrophobia; (6) subjects with mental disorders.

The baseline data of participants, including demographic information, were recorded. Patients were instructed how to install and use the App and started self-management of the Telehealth-based monitoring system.

### Monitoring acute exacerbation of COPD

**Fig. 1:**
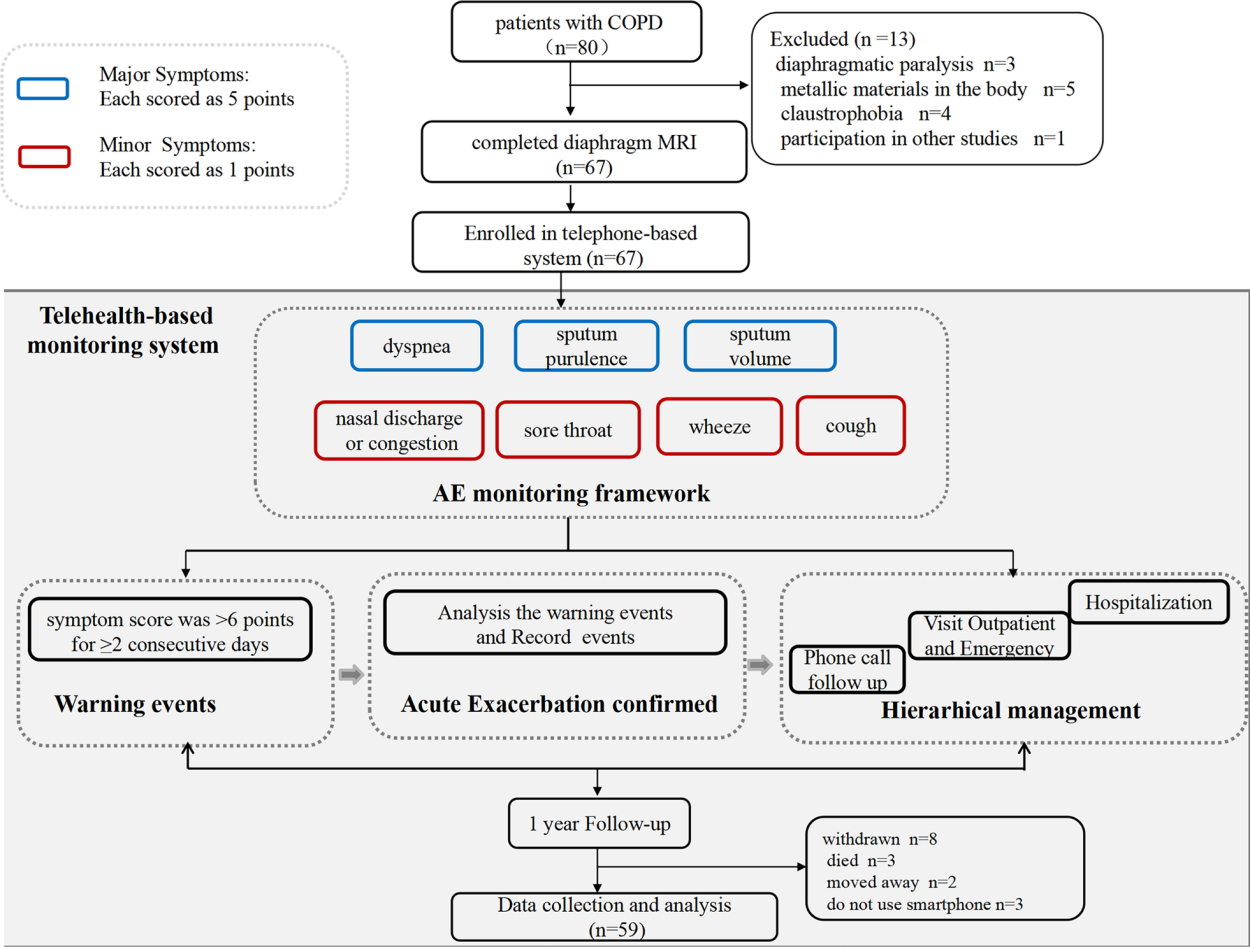
Flow diagram of patients’ recruitment and AE monitoring based on Telehealth-based monitoring system. COPD, chronic obstructive pulmonary disease; MRI, magnetic resonance imaging; AE, acute exacerbation

The acute exacerbation events monitoring system was developed in collaboration with Zhejiang University, as described in our previously published paper [26]. The system was used for acute exacerbation risk evaluation, disease management, risk warning, follow-up, and compliance management. The online diary allows patients to track their discomfort symptoms through the app, while the workstation automatically detects acute exacerbations. Definition of acute exacerbations based on a previously validated criterion [27]. The system automatically detected a suspected exacerbation if the summed symptom score was > 6 points for ≥ 2 consecutive days. The detection required further confirmation by health care providers to ensure whether the symptoms were caused by COPD. The Telehealth-based monitoring system is illustrated below in Fig. 1.

### MRI acquisition and analysis

**Fig. 2:**
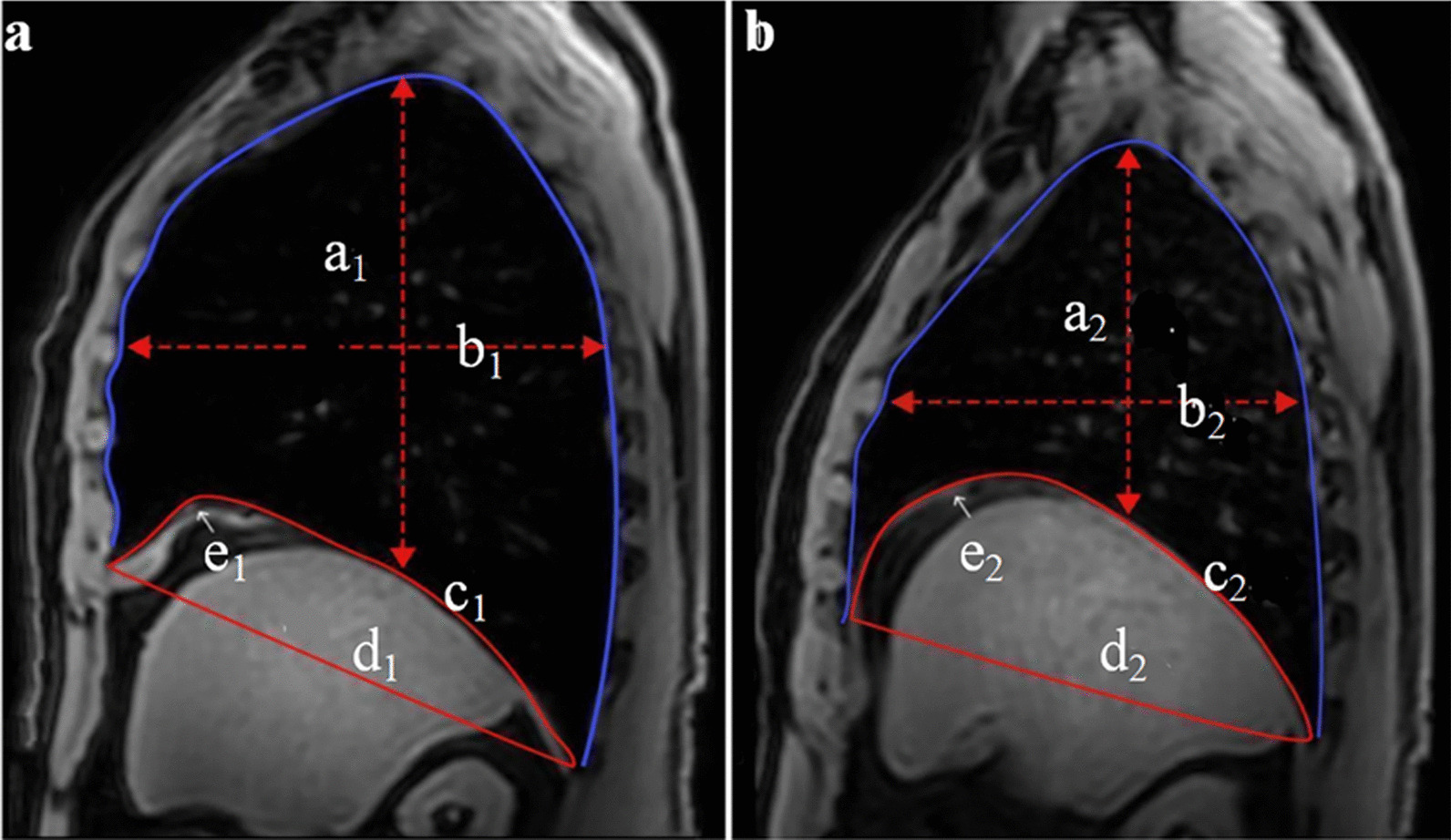
Methods for measuring diaphragm function parameters at the end of maximal breathing. The image shows a single frame of the right sagittal magnetic resonance imaging data for one participant. The lung area, which is the area inside the lung contour (coloured blue) at the end of inspiration (**a**) and expiration (**b**). a_1_, a_2_ present the upper-lower diameters, which was the vertical distance between the lung apex and hemidiaphragm is measured at end of inspiration (a_1_) and expiration (a_2_), and the two dimensions are subtracted to obtain the diaphragmatic displacement by length (a_1_–a_2_). b_1_, b_2_ present the anterior–posterior diameter of the chest, which was the trans-vertical anteroposterior dimension of the upper and lower thoraces at the right mid-sagittal planes measured at end of expiration (b_2_) and inspiration (b_1_), and the two dimensions are subtracted to obtain the chest wall motion (b_1_–b_2_). The white arrow shows the diaphragm thickness (DT) at the end of maximal inspiration (e_1_) and expiration (e_2_). c_1_ presents the diaphragm length, which is the length across the diaphragmatic dome from the anterior to the posterior chest wall margin at the end of maximal inspiration. c_2_ presents it at the end of maximal expiration; d1 presents the linear distance of the anteroposterior costophrenic angle at the end of maximal inspiration; d_2_ presents it at the end of maximal expiration. Diaphragmatic dome factor (ins) = c_1_/d_1_. Diaphragmatic dome factor (exp) = c_2_/d_2_. Diaphragm thickening fraction (DTF) = (DT at end of maximal inspiration) – (DT at end of maximal expiration)]/ (DT at end of maximal expiration) × 100%

MRI examinations were performed with subjects lying supine inside a 3.0 T MRI system (Philips Medical Systems, Ingenia, The Netherlands) with a 64-channel coil. We used the sequence of 3D mDIXON SENSE. The field of view (FOV) was 360 mm, the matrix size was 512*512, and the image slice thickness was 3 mm. Images of the lung and diaphragm were obtained during eupnoea and at the end of maximal breathing. In order to ensure that all breathing maneuvers are executed correctly, and with maximum effort, all breathing maneuvers are trained and instructed by an experienced physician before and during the MRI, and they did not use sedatives or contrast agents. To ensure get the best results for the MRI examination, the patients were advised to breathe deeply twice under the coil before the images were scanned. Each participant was encouraged to repeatedly breathe deeply and regularly for five to seven respiratory cycles from maximal inspiration to maximal expiration with as much effort as possible [28]. The acquisition time of a patient is approximately 20 to 25 min. Subjects were instructed to breathe in and out deeply upon hearing the scanner alarming(respiratory rate of 6 to 8 breaths per minute). Furthermore, the rest of the time, their respiratory rate of breath is normal. Images were checked by the radiologist and reacquired during the same imaging session if unsatisfactory. During MRI examination, 13 patients were unable to tolerate in a supine position (2 had dizziness, 5 had pain in the limbs, 2 had dyspnea, and 4 had claustrophobia) in our study. We paused the process immediately and allow the patient to relax. All of them felt better and continue to acquire images after the rest. If the patient refuses collection, we will terminate the acquisition, and the patient will withdraw from the study. According to previous studies, the parameters of diaphragmatic dome factor [29], diaphragmatic displacement [30], chest wall motion [28], lung area, and diaphragm thickness were ascertained and manually measured by two or more experienced radiologists at the end of maximal breathing. The measuring method is shown in Fig. 2, and the diaphragm function data are showed in Additional file 1.

### Statistical analysis

The data were analysed by SPSS version 26.0 for Windows (IBM Corp, Armonk, New York, USA). Continuous variables with normal distribution were presented as mean ± standard deviation (SD). Non-normally distributed variables were reported as median (inter-quartile range). A t-test was used to compare variables between the COPD group and healthy control group, as well as between the frequent AECOPD group and the infrequent AECOPD group. ANOVA was used to analyse the within-group difference between the GOLD stage. Pearson or Spearman correlation test was applied to assess the relationship of variables. Univariate and multivariable logistic regression analyses were applied to identify the risk factors for the frequency of AECOPD. The area under the receiver operating characteristic curve (ROC) was applied to compare the prediction ability for the frequency of AECOPD risk according to diaphragm function parameters with different thresholds. A value of *P* < 0.05 was considered significant in all statistical analyses.

## Results

### Demographics of study subject

Of the 80 COPD patients, of which 59 patients had one year of complete prospective follow-up data, 21 were excluded for various reasons, including three patients were excluded due to diaphragmatic paralysis, five patients with metallic materials in the body, four with claustrophobia, and one participating in other studies. However, after one year of follow-up, eight patients withdrew from the study, including three died, two moved to other cities, and three failed to use smartphones properly. The COPD group included 56 males and 3 females with a mean age of 60.37 ± 6.56 years; 9 COPD patients were categorized as mild, 19 as moderate, 18 as severe, and 13 as very severe based on respiratory function. A total of 20 healthy controls were enrolled, and 16 completed the study; the mean age was 58.75 ± 7.10, including 11 males and 5 females. Gender, smoking status, and the pulmonary function parameters, FEV1, FEV1% predict, FVC, FVC% predict, and FEV1/FVC% were significantly different between the COPD group and healthy controls (*P* < 0.05). Patient demographics and clinical characteristics are shown in Table 1.

**Table 1:** The baseline characteristics between the COPD group and healthy controls

Various	COPD group (N = 59)	Healthy control (N = 16)	*t/X*^2^	*P *value
*Demographic*				
Sex (male/female)	56/3	11/5	9.04^a^	0.003
Age (years)	60.37 ± 6.56	58.75 ± 7.10	0.86	0.391
BMI (kg/cm^2^)	24.23 ± 2.84	23.61 ± 3.53	0.74	0.465
*Smoking (n (%))*				
Ex-smoker	37 (62.71%)	0	–	–
Current smoker	14 (23.73%)	0	–	–
Non-smoker	8 (13.56%)	16 (100%)	43.22^a^	*P* < 0.001
*Pulmonary function*				
FEV1 (L)	1.68 ± 0.91	2.69 ± 0.45	− 4.28	*P* < 0.001
FEV1, %predicted	51.34 ± 22.24	89.82 ± 13.51	− 6.58	*P* < 0.001
FVC(L)	2.93 ± 0.93	3.17 ± 0.58	− 0.96	0.340
FVC, %predicted	74.09 ± 21.25	86.93 ± 13.30	− 2.29	0.005
*COPD severity, n (%)*				
GOLD1	9(15.25%)	–	–	–
GOLD2	19(32.20%)	–	–	–
GOLD3	18(30.51%)	–	–	–
GOLD4	13(22.03%)	–	–	–
*Self*-*management*				
CAT scores	13.80 ± 6.45	–	–	–
mMRC	1 (1,3)			
6MWT(m)	447.07 ± 102.43	–	–	–

Data are expressed as means ± SD, number or percentage are expressed as n (%), except where otherwise noted. a. present chi-square test

COPD, chronic obstructive pulmonary disease; BMI, body mass index; FEV1, forced expiratory volume at 1 s; FVC, forced vital capacity; GOLD, global initiative for chronic obstructive lung disease; CAT, chronic obstructive pulmonary disease assessment test; mMRC, modified Medical Research Council; 6MWT, 6-min walk test; SD, standard deviation

### Comparison of diaphragm function between COPD group and healthy group

**Fig. 3:**
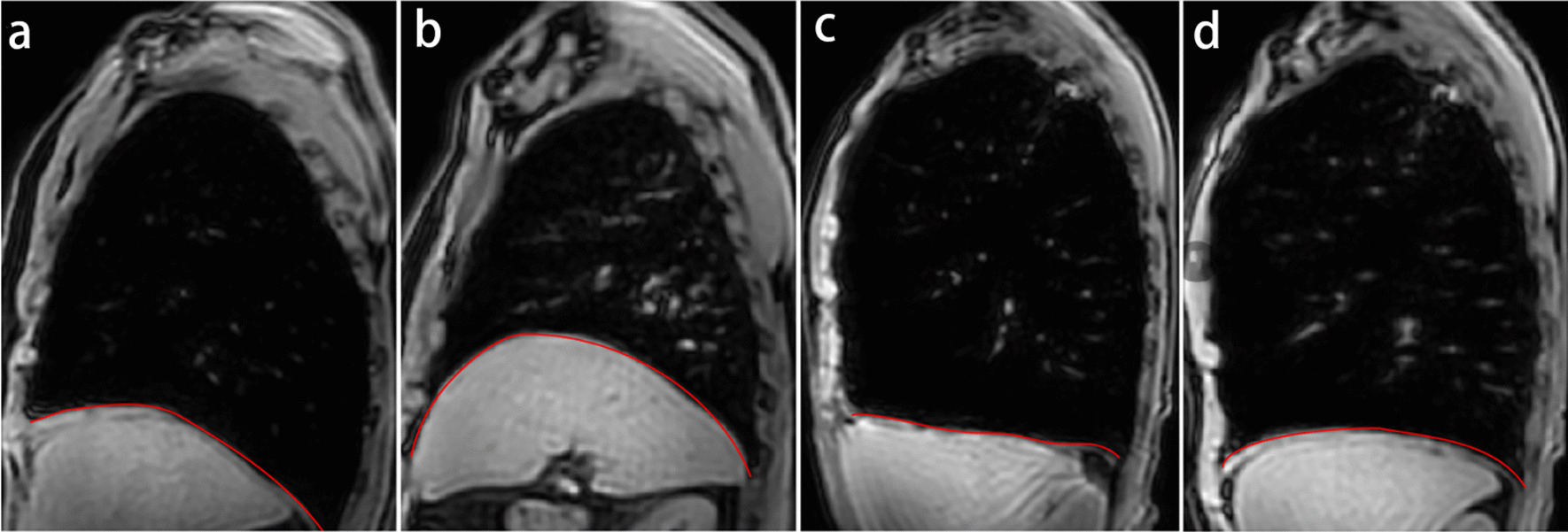
The morphology of diaphragm and lung hyperinflation at the end of maximal breathing. a,b. Describe the structure and function of the diaphragm and their lung hyperinflation in a 65-year-old healthy male; c.d. Describe the morphology of diaphragm and lung hyperinflation in a 65-year-old man with severe COPD (FEV1%predict 20.83). In the healthy control, the lung size and location of the diaphragm are obviously changed from inspiration (**a**) to expiration (**b**); however, in the COPD patient, the lung size and location of the diaphragm are not largely changed from inspiration (**c**) to expiration (**d**), the diaphragm becomes flat and short, suggesting the presence of severe air-trapping and airflow limitation. Thus, COPD patients have dysfunction of the diaphragm.

The parameters of diaphragm function were measured by MRI on the right side, both during the end of maximal inspiration and expiration. The diaphragmatic dome factor was lower in the COPD group than in the healthy control group at the end of maximal breathing (1.15 ± 0.09 vs. 1.27 ± 0.11, *P* < 0.001; 1.24 ± 0.13 vs. 1.32 ± 0.07, *P* = 0.02). Anterior–posterior diameter of the chest at the end of maximal inspiration and expiration was higher in the COPD group than in the healthy control group (18.78 ± 1.61 vs. 17.20 ± 1.98, *P* = 0.001; 16.88 ± 1.64 vs. 14.31 ± 1.92, *P* < 0.001). Chest wall motion was lower in the COPD group than in the healthy control group (1.91 ± 1.07 vs. 2.89 ± 1.38, *P* = 0.003). Lung area at the end of maximal inspiration and expiration were higher in the COPD group than in the healthy control group (355.25 ± 43.62 vs. 305.59 ± 42.17, 244.28 ± 52.94 vs. 172.28 ± 29.04, respectively, *P* < 0.001). The change of lung area was lower in the COPD group than in the healthy control group (110.98 ± 40.30 vs. 133.31 ± 36.50, *P* = 0.049). Upper-lower diameter of the lung at the end of maximal inspiration and expiration was higher in patients with COPD than in the healthy subjects (23.15 ± 2.48 vs. 21.80 ± 1.49, *P* = 0.009, 19.00 ± 3.49 vs. 14.56 ± 1.45, *P* < 0.001). Diaphragmatic displacement was lower in the COPD group than in the healthy control group (4.16 ± 2.02 vs. 7.24 ± 1.26, *P* < 0.001). Diaphragm thickness at the end of maximal inspiration and the diaphragm thickening fraction (DTF) were lower in the COPD group than in the healthy control group (6.04 ± 1.07 vs. 7.04 ± 1.02, *P* = 0.001; 29.08 ± 16.57 vs. 66.11 ± 22.46, *P* < 0.001). The diaphragm thickness at the end of maximal expiration was thicker in the COPD group than in the control group (4.69 ± 0.61 vs. 4.30 ± 0.78, *P* = 0.038). The results presented in Table 2. Figure 3 clearly shows the diaphragm morphology and lung hyperinflation at the end of maximal inspiration and expiration in a healthy control and a COPD patient.

**Table 2:** Analysis of diaphragm function parameters between COPD and control groups

Variables	COPD group (N = 59)	Healthy control (N = 16)	*t*	*P* value
*Diaphragmatic dome factor*				
Diaphragmatic dome factor (Insp)	1.15 ± 0.09	1.27 ± 0.11	− 4.42	*P* < 0.001
Diaphragmatic dome factor (Exp)	1.24 ± 0.13	1.32 ± 0.07	− 2.38	0.020
*Anterior–posterior diameter of chest(cm)*				
Anterior–posterior diameter Insp(cm)	18.78 ± 1.61	17.20 ± 1.98	3.33	0.001
Anterior–posterior diameter Exp(cm)	16.88 ± 1.64	14.31 ± 1.92	5.37	*P* < *0.001*
Chest wall motion(cm)	1.91 ± 1.07	2.89 ± 1.38	− 3.07	0.003
*Lung area (cm*^*2*^*)*				
Lung area Insp (cm^2^)	355.25 ± 43.62	305.59 ± 42.17	4.07	*P* < *0.001*
Lung area Exp (cm^2^)	244.28 ± 52.94	172.28 ± 29.04	7.19	*P* < *0.001*
Change of lung area (cm^2^)	110.98 ± 40.30	133.31 ± 36.50	− 2.00	0.049
*Upper-lower diameter of lung (cm)*				
Upper-lower diameter Insp (cm)	23.15 ± 2.48	21.80 ± 1.49	2.74	0.009
Upper-lower diameter Exp (cm)	19.00 ± 3.49	14.56 ± 1.45	7.65	*P* < *0.001*
Diaphragmatic displacement (cm)	4.16 ± 2.02	7.24 ± 1.26	− 5.81	*P* < *0.001*
*Diaphragm thickness (cm)*				
Thickness Insp (cm)	6.04 ± 1.07	7.04 ± 1.02	− 3.34	0.001
Thickness Exp (cm)	4.69 ± 0.61	4.30 ± 0.78	2.11	0.038
Diaphragm thickening fraction	29.08 ± 16.57	66.11 ± 22.46	− 7.32	*P* < *0.001*

Data are expressed as means ± SD except where otherwise noted

COPD, chronic obstructive pulmonary disease; Insp, inspiration; Exp, expiration; SD, standard deviation

### Comparison of diaphragm function between COPD subgroup

**Fig. 4:**
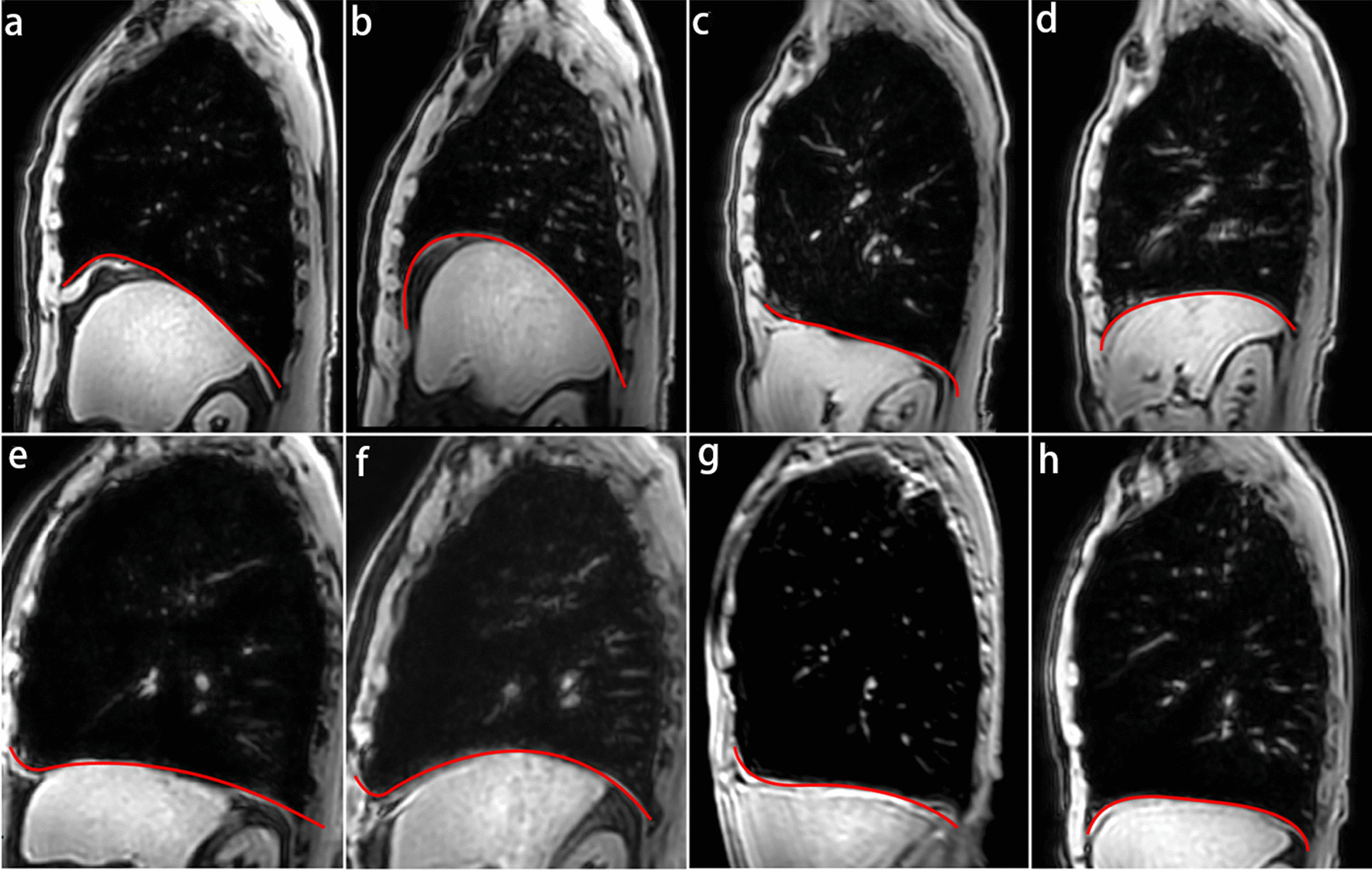
The morphology of diaphragm and lung hyperinflation at end of maximal breathing in COPD subgroups. a.b. present mild group at the end of maximal inspiration (**a**) and expiration (**b**); c.d. present moderate group at the end of maximal inspiration (**c**) and expiration (**d**); e.f. present severe group at the end of maximal inspiration (**e**) and expiration (**f**); g.h. present very severe group at the end of maximal inspiration (**g**) and expiration (**h**). Mild group, FEV1 ≥ 80% predicted; moderate group, 50% ≤ FEV1 < 80% predicted; severe group, 30% ≤ FEV1 < 50% predicted; very severe group, FEV1 < 30% predicted

COPD patients were classified into four groups according to the GOLD stages: 9 (15.25%) patients in the mild group, 19 (32.20%) in the moderate group, 18 (30.51%) in the severe group, and 13 (22.03%) in the very severe group, respectively. As presented in Table 3 (at the end of the document text file), ANOVA analysis revealed significant differences in the lung area, the upper-lower diameter at the end of maximal inspiration and expiration, the chest wall motion, the change of lung area, the diaphragmatic displacement, the diaphragm thickening fraction, and the diaphragm thickness at the end of maximal inspiration, among patients with different severity of COPD (all *P* < 0.05). But no statistical differences were found in the diaphragmatic dome factor, the anterior–posterior diameter of the chest at the end of maximal inspiration and expiration, and diaphragm thickness at the end of maximal expiration among four groups (all *P* > 0.05). Figure 4 clearly showed the diaphragm morphology and the lung hyperinflation in COPD patients with varied severities. As the respiratory function deteriorates, the diaphragm morphology appeared to be more flattened, and lung hyperinflation becomes more pronounced.

**Table 3:** Comparison of diaphragm function parameters between COPD subgroup at the end of maximal breathing

Variables	Mild group (N = 9)	Middle group (N = 19)	Severe group (N = 18)	Very severe group (N = 13)	*F*	*P*
*Diaphragmatic dome factor*						
Insp	1.20 ± 0.07^✭^	1.16 ± 0.11	1.15 ± 0.09	1.10 ± 0.04	2.39	0.079
Exp	1.28 ± 0.09^✭^	1.24 ± 0.04	1.28 ± 0.19^◇^	1.16 ± 0.11	2.77	0.050
*Anterior–posterior diameter of chest (cm)*						
Anterior–posterior diameter Insp (cm)	19.52 ± 1.86	18.83 ± 1.33	18.33 ± 1.78	18.82 ± 1.51	1.11	0.353
Anterior–posterior diameter Exp (cm)	16.34 ± 1.83^✭^	16.99 ± 1.37	16.36 ± 1.86^◇^	17.79 ± 1.21	2.50	0.069
Chest wall motion (cm)	3.18 ± 1.31^✭✭✭^	1.84 ± 0.90***	1.97 ± 0.69^##◇◇^	1.02 ± 0.64^△^	11.04	*P* < 0.001
*Lung area (cm*^*2*^*)*						
Lung area Insp (cm^2^)	355.01 ± 21.52	346.53 ± 44.02^◇◇^	342.58 ± 40.29	385.72 ± 48.13^△^	3.20	0.030
Lung area Exp (cm^2^)	190.53 ± 32.04^✭✭✭^	226.01 ± 36.03*	245.53 ± 37.70^##◇◇◇^	306.45 ± 44.88^△△△^	18.96	*P* < 0.001
Change of lung area (cm^2^)	164.48 ± 23.24^✭✭✭^	120.52 ± 28.33**	97.06 ± 29.04^###□^	79.27 ± 37.72^△△△^	16.10	*P* < 0.001
*Upper-lower diameter of the lung (cm)*						
Upper-lower diameter Insp (cm)	21.89 ± 1.42^✭✭✭^	22.16 ± 2.46	22.73 ± 1.76^◇◇◇^	26.07 ± 1.64^△△△^	12.81	*P* < 0.001
Upper-lower diameter Exp (cm)	14.55 ± 1.78^✭✭✭^	16.57 ± 2.22**^□□^	18.80 ± 1.99^###◇◇◇^	22.81 ± 1.49^△△△^	27.39	*P* < 0.001
Diaphragmatic displacement (cm)	7.34 ± 1.74^✭✭✭^	5.58 ± 1.74*	3.94 ± 1.28^###□□^	3.26 ± 1.05^△△△^	13.32	*P* < 0.001
*Diaphragm thickness (mm)*						
Thickness Insp (mm)	6.44 ± 1.00^✭^	6.47 ± 1.35	5.83 ± 0.74	5.42 ± 0.71	3.51	0.021
Thickness Exp (mm)	4.61 ± 0.71	4.64 ± 0.59	4.79 ± 0.61	4.66 ± 0.62	0.26	0.852
Diaphragm thickening fraction	40.51 ± 15.82^✭✭✭^	38.50 ± 15.68	22.54 ± 14.31^##□□^	16.45 ± 5.18^△△△^	10.22	*P* < 0.001

Data are presented as mean ± SD unless otherwise stated

Mild group: FEV1 ≥ 80% predicted; moderate group: 50% ≤ FEV1 < 80% predicted; severe group: 30% ≤ FEV1 < 50% predicted; very severe group: FEV1 < 30% predicted. *P* values derived from ANOVA testing. *, **, *** comparison between mild and moderate (*P*˂0.05, *P*˂0.01, *P*˂0.001); ^#^, ^##^, ^###^ comparison between mild and severe (*P*˂0.05, *P*˂0.01, *P*˂0.001); ^✭^, ^✭✭✭^ comparison between mild and very severe (*P*˂0.05, *P*˂0.001), ^□^ comparison between moderate and severe (*P*˂0.05); ^△^, ^△△^, ^△△△^comparison between moderate and very severe (*P*˂0.05, *P*˂0.01, *P*˂0.001); ^◇^, ^◇◇^, ^◇◇◇^comparison between severe and very severe (*P*˂0.05, *P*˂0.01, *P*˂0.001)

### Correlation analysis

**Fig. 5:**
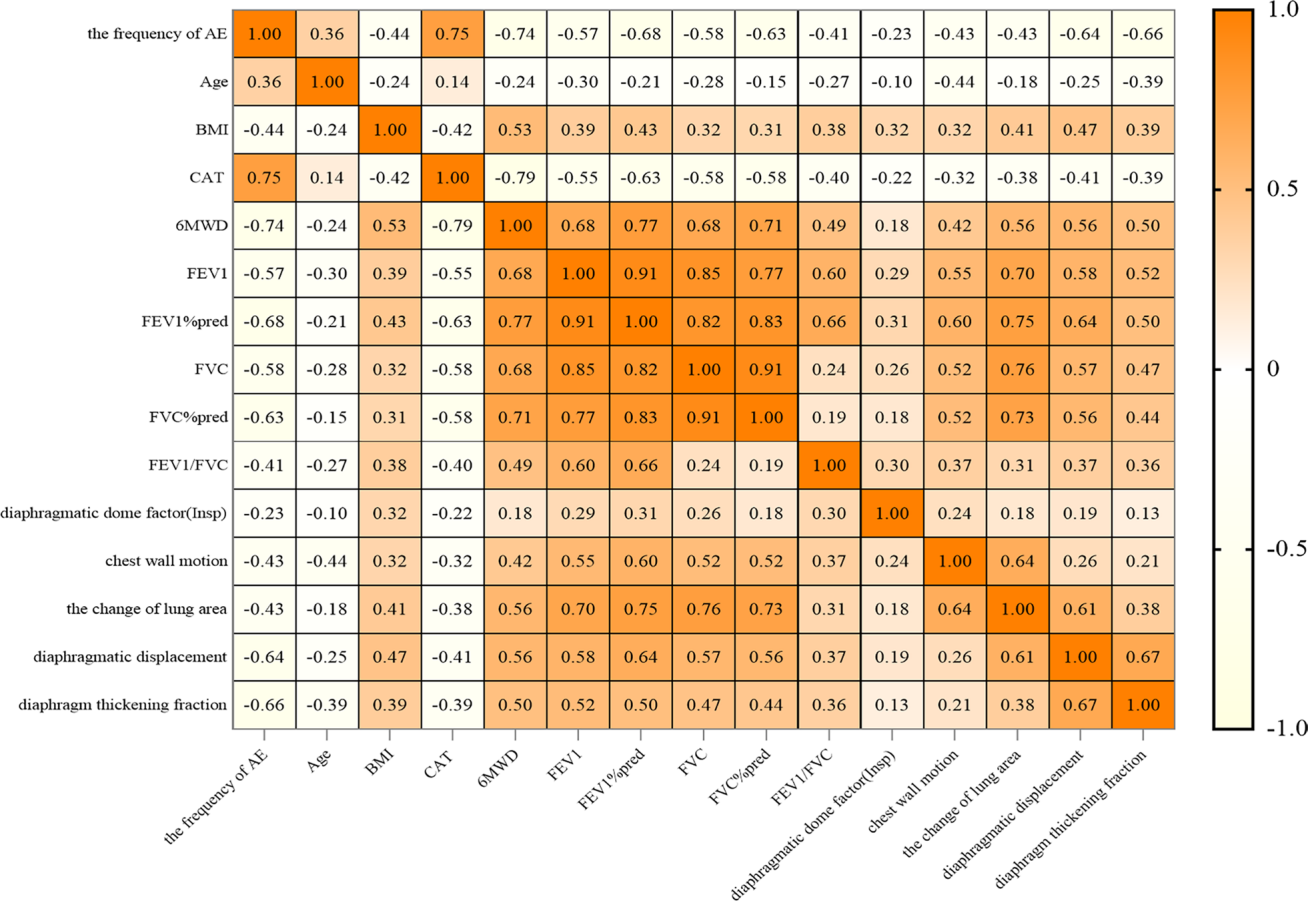
Correlation matrix of clinical parameters and diaphragm function. AE, acute exacerbation; BMI, body mass index; CAT, chronic obstructive pulmonary disease assessment test; 6MWT, 6-min walk test; FEV1, forced expiratory volume at 1 s; FVC, forced vital capacity

Correlation matrix analyses were conducted between clinical parameters and diaphragm function data. The results were presented in Fig. 5 and Additional file 2. A matrix graph of Pearson's correlation analysis indicated that the diaphragm function parameters (the chest wall motion, change of lung area, diaphragmatic displacement, and diaphragm thickening fraction) are closely related to pulmonary function parameters (FEV1, FEV1% pred, FVC, and FVC% pred) and 6MWD with a correlation coefficient between 0.4 and 0.8. In addition, a negative degree of correlation was observed among the diaphragm function parameters, the frequency of AECOD, and CAT scores (correlation coefficient between 0.3 and 0.7).

### Monitoring of AECOPD

**Fig. 6:**
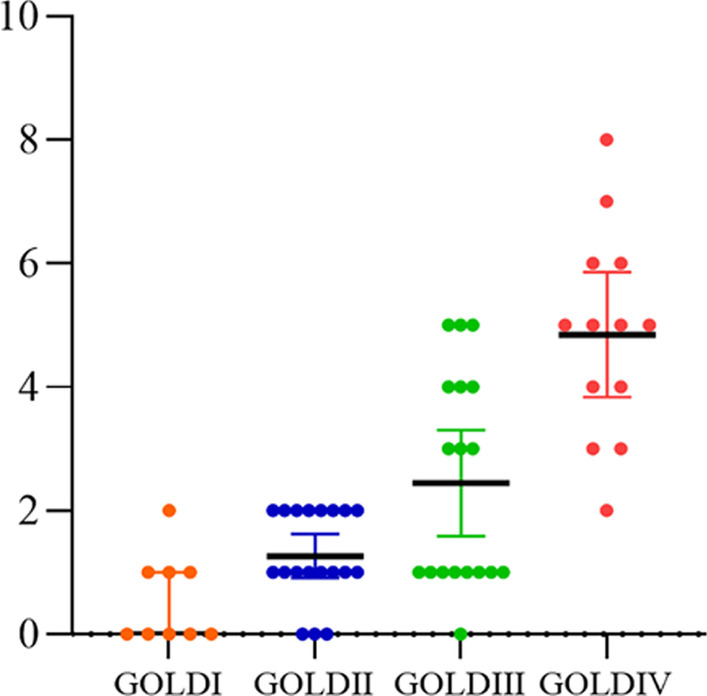
Exacerbations of COPD in different subgroups over the course of a one-year follow-up. GOLD: Global Initiative for Chronic Obstructive Lung Disease; GOLD I: FEV1 ≥ 80% predicted; GOLD II: 50% ≤ FEV1 < 80% predicted; GOLD III: 30% ≤ FEV1 < 50% predicted; GOLD IV: FEV1 < 30% predicted

Under the Telehealth-based monitoring system, a total of 719 warning events were recorded. Among them, 136 warning events resulted from acute exacerbations of COPD following a one-year follow-up period. Twenty-eight patients experienced acute exacerbations of less than two episodes, including nine patients who did not experience any acute exacerbation. Thirty-one patients experienced two or more exacerbations in the one-year follow-up. The average frequency of acute exacerbation was 2.31 times. As shown in Fig. 6, the frequency of acute exacerbations in COPD patients increased with GOLD grade and it is significantly higher for patients with GOLD 3 (severe) and GOLD 4 (very severe). After one year of follow-up, among GOLD I patients, one patient had episode two acute exacerbations, with a median of 0, IQR (0–1); among GOLD II patients, eight patients had episode two acute exacerbations, with a median of 1, IQR (1, 2); nine patients had two or more acute exacerbations in GOLD III, with a median of 2, IQR (1,4); Remarkably, all the patients had experienced at least two acute exacerbations in the GOLD IV group, the median was 5, IQR (4,6).

### Comparison of diaphragm function between frequent AECOPD group and infrequent AECOPD group

According to the frequency of AECOPD, patients with COPD were divided into two groups: the infrequent AECOPD group (defined as less than two episodes of exacerbation per year) and the frequent AECOPD group (defined as two or more exacerbations per year). Compared with the infrequent AECOPD group, the frequent AECOPD group showed older age (*P* = 0.005), lower BMI (*P* = 0.001), higher CAT scores (*P* = 0.001), more severe mMRC (*P* = 0.001), a shorter distance of 6MWD (*P* < 0.001), worse lung function (EFV1:*P* < 0.001; FEV1% pred: *P* < 0.001; FVC: *P* = 0.007; FVC% pred: *P* = 0.001), lower chest wall motion (*P* = 0.002), lower change of lung area (*P* = 0.003), lower diaphragmatic displacement (*P* < 0.001), and significantly decreased diaphragm thickening fraction (*P* < 0.001). No significant differences were found in the diaphragmatic dome factor at the end of maximal inspiration and expiration. Overall, these results suggest that patients in the frequent AECOPD group had worse pulmonary function status, health status, and diaphragm function than those in the infrequent AECOPD group (Table 4).

**Table 4:** Baseline characteristics and diaphragm function parameters of frequent and infrequent AECOPD patients

Variables	Infrequent AE (N = 28)	Frequent AE (N = 31)	*t/Z*	*P*
Age (year)	57.89 ± 6.94	62.61 ± 5.37	− 2.94	0.005
BMI (kg/cm^2^)	25.46 ± 2.56	23.11 ± 2.64	3.47	0.001
CAT score	10.89 ± 3.04	16.42 ± 7.55	− 3.62	0.001
mMRC	1 (1,1)	2 (1,3)^a^	− 3.19	0.001
6MWD(m)	500.07 ± 60.66	399.19 ± 109.46	4.31	*P* < 0.001
FEV1(L)	2.11 ± 0.95	1.30 ± 0.67	3.82	*P* < 0.001
FEV1/%pre	63.41 ± 20.27	40.44 ± 18.11	4.60	*P* < 0.001
FVC(L)	3.27 ± 0.81	2.63 ± 0.94	2.80	0.007
FVC/%pre	83.22 ± 16.40	65.85 ± 21.97	3.41	0.001
GOLDI	8 (28.57%)	1 (3.23%)	–	–
GOLDII	11 (39.29%)	8 (25.81%)	–	–
GOLDIII	9 (32.14%)	9 (29.03%)	–	–
GOLDIV	0 (0%)	13 (41.94%)	–	–
Diaphragmatic dome factor (Insp)	1.17 ± 0.10	1.13 ± 0.07	1.65	0.105
Diaphragmatic dome factor (Exp)	1.25 ± 0.07	1.23 ± 0.17	0.71	0.480
Chest wall motion(cm)	2.35 ± 1.05	1.51 ± 0.93	3.25	0.002
Change of lung area (cm^2^)	126.71 ± 37.79	96.76 ± 37.62	3.05	0.003
Diaphragmatic displacement (cm)	5.33 ± 1.99	3.10 ± 1.36	4.98	*P* < 0.001
Diaphragm thickening fraction	39.83 ± 16.25	19.36 ± 9.38	5.84	*P* < 0.001

Data are expressed as means ± SD, number or percentage, except where otherwise states

AECOPD, acute exacerbations of chronic obstructive pulmonary disease; BMI, body mass index; CAT, chronic obstructive pulmonary disease assessment test; mMRC, modified Medical Research Council; 6MWT, 6-min walk test. FEV1, forced expiratory volume at 1 s; FVC, forced vital capacity; GOLD, global initiative for chronic obstructive lung disease; Insp, inspiration; Exp, expiration

^a^Mann–Whitney U-test. Frequent AECOPD is defined as two or more exacerbations per year. Infrequent AECOPD is defined as less than two episodes of exacerbation per year

### Predictive factors for frequency of AECOPD during a one-year follow-up in patients with COPD

Variables with statistical significance in comparisons of the clinical parameters and diaphragm function data were included in the multivariate logistic regression analysis. The backward stepwise regression method was adopted. Eleven variables were significantly associated with an increased risk of frequent AECOPD on univariate analysis and entered in the logistic regression model: age (*P* = 0.009), BMI (*P* = 0.003), CAT scores (*P* = 0.003), 6MWD.

(*P* = 0.001), FEV1% pred (*P* < 0.001), FVC% pred (*P* = 0.004), FEV1/FVC% (*P* = 0.007), chest wall motion (*P* = 0.032), the change of the lung area (*P* = 0.007), diaphragmatic displacement (*P* < 0.001), and the diaphragm thickening fraction (*P* < 0.001). After further adjusting for covariates, the chest wall motion (OR 0.37, 95% CI: 0.15–0.92, *P* = 0.032) and the diaphragm thickening fraction (OR: 0.89, 95% CI 0.83–0.94, *P* < 0.001) were independently associated with the frequency of AECOPD (Table 5).

**Table 5:** Logistic regression analysis of risk factors associated with the frequency of AECOPD

	Univariate analysis			Multivariate analysis		
	OR	95% CI	*P*	OR	95% CI	*P*
Age	1.14	1.03–1.25	0.009			
BMI	0.70	0.56–0.89	0.003			
CAT scores	1.19	1.06–1.34	0.003			
6MWD(m)	0.99	0.98–1.00	0.001			
FEV1%pred	0.94	0.91–0.97	0.000			
FVC%pred	0.95	0.92–0.99	0.004			
FEV1/FVC%	0.93	0.89–0.98	0.007			
Diaphragmatic dome factor (Insp)	0.01	0.00–4.56	0.125			
Diaphragmatic dome factor (Exp)	0.22	0.00–16.10	0.485			
Chest wall motion(cm)	0.37	0.15–0.92	0.032	0.37	0.15–0.92	0.032
Change of lung area (cm^2^)	0.979	0.96–0.99	0.007			
Diaphragmatic displacement (cm)	0.45	0.29–0.69	0.000			
Diaphragm thickening fraction	0.88	0.83–0.94	0.000	0.89	0.89–0.94	0.000

ECOPD, acute exacerbations of chronic obstructive pulmonary disease; BMI, body mass index; CAT, chronic obstructive pulmonary disease assessment test; 6MWT, 6-min walk test; FEV1, forced expiratory volume at 1 s; FVC, forced vital capacity; OR odds ratio; 95% CI 95% confidence interval; Insp, inspiration; Exp, expiration

### ROC analysis of the predictive values of the simple and combined index

**Fig. 7:**
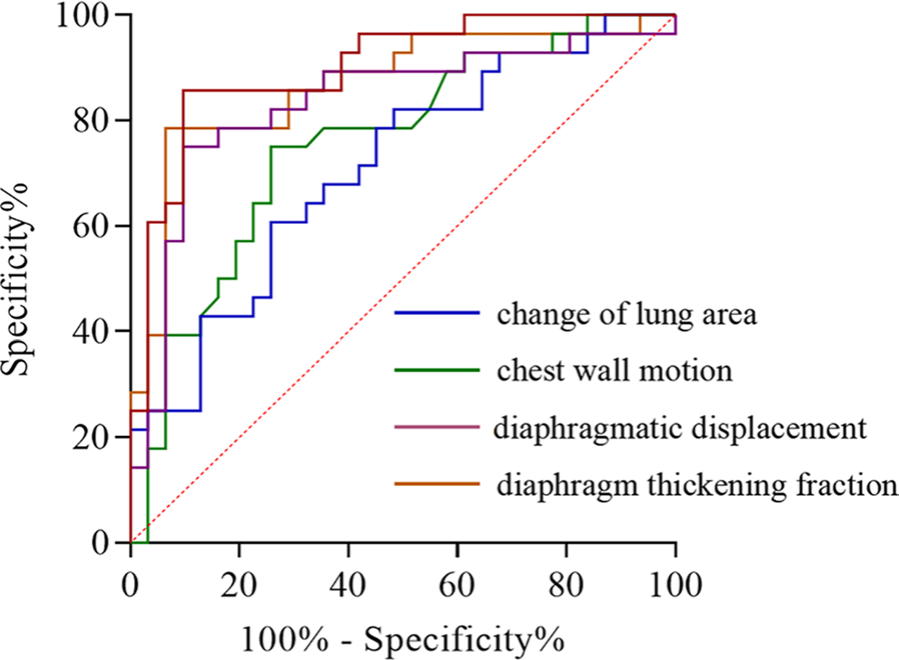
Receiver operating characteristic curves of diaphragm function indices predicting the frequency of AECOPD. The area under the curve (AUC) of the change of lung area during maximal deep breathing was 0.756. The AUC of chest wall motion during maximal deep breathing was 0.711. The diaphragmatic displacement during maximal deep breathing was 0. 833. The diaphragm thickening fraction during maximal deep breathing was 0.869. AECOPD, acute exacerbation of chronic obstructive pulmonary disease; AUC, area under the curve

**Fig. 8:**
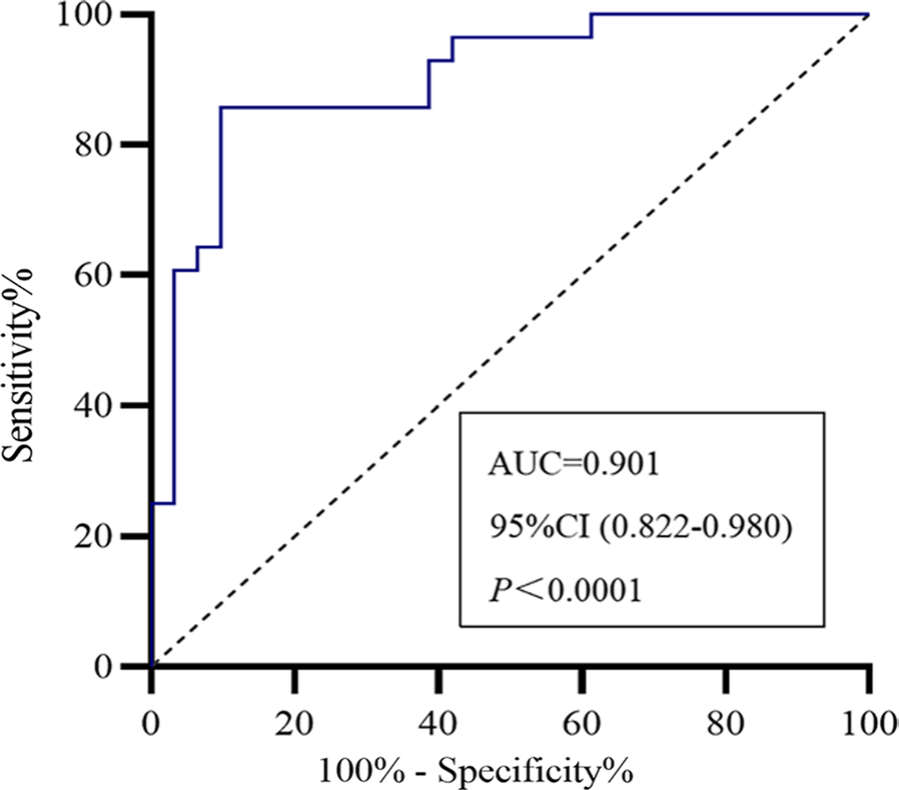
Receiver operating characteristic curves for risk factors of combined diaphragm function indexes in predicting AECOPD. The AUC of chest wall motion combined with the diaphragm thickening fraction was 0.901, 95% CI (0.822–0.980), *P* < 0.001. The sensitivity and specificity were 90.3% and 83%, respectively. AECOPD, acute exacerbation of chronic obstructive pulmonary disease; AUC, area under the curve; CI, confidence interval

A ROC analysis was conducted to determine the optimal cut-off values of the diaphragm function to predict the frequency of AECOPD. The sensitivity, specificity, optimal threshold, and AUC for the chest wall motion, change of lung area, diaphragmatic displacement, and diaphragm thickening fraction were shown in the Additional file 3; corresponding ROC curves are presented in Fig. 7. As far as predicted ability was concerned, all four parameters were suitable, but the diaphragm thickening fraction has the largest AUC 0.869 (95% CI: 0.722–0.965). Moreover, the optimal cut-off value was 29.51% at a sensitivity of 78.6%, a specificity of 93.5%. We also found that the diaphragm thickening fraction was combined with chest wall motion to predict the frequency of AECOPD has the best AUC, sensitivity and specificity index (AUC = 0.901, 95% CI: 0.822–0.980, *P* < 0.0001, sensitivity: 90.3%, specificity: 85.7%) (Fig. 8).

## Discussion

In this study with small size of sample, we investigated whether the diaphragm parameters could predict the acute exacerbations of AECOPD. Results showed that the diaphragmatic functional parameters, especially chest wall motion and diaphragmatic thickening fraction. These indexes could be used for the prediction of the frequency of acute exacerbation of AECOPD, and could further be potential risk factors for clinically stable COPD patients that occur the frequency of acute exacerbations in the future. To our knowledge, we first proved evidence that the inclusion of DD can aid to predict AECOPD. This study thus also offers a novel index to make up a lack of other conventional indicators in predicting AECOPD in COPD patients.

Our results show that in the frequent AECOPD group, patients have severe airflow limitation (FEV1%pred:40.44 ± 18.11), low exercise capacity (6MWD:399.19 ± 109.46 m), and moderate disease burden (CAT score: 16.42 ± 7.55) and poor diaphragm dysfunction (seen in Table 4). Therefore, for patients who are at risk of frequent acute exacerbations, we suggested to undergo the diaphragmatic MRI to clarify the functional status of the diaphragm and provide targeted interventions to prevent acute exacerbation events. However, considering the cost-effectiveness, the clinically indicated patients with low disease burden, breathlessness, good exercise capacity, and moderate disease (FEV1 over 50) may not request to complete the MRI unless the patient who is particularly susceptible to exacerbations risks.

In addition, we also explored the relationship between diaphragmatic function parameters, disease severity, and acute exacerbation frequency. The results of our study showed that diaphragm dysfunction occurs in COPD patients, mainly characterized by a short and flat diaphragm dome, hyperinflation of the lung area, chest wall motion, diaphragmatic displacement, and the diaphragm thickening fraction, which were positively correlated with COPD severity.

To our knowledge, COPD is a heterogeneous disorder characterized by small airway dysfunction and parenchymal and vascular destruction [31]. Extensive studies on COPD patients have demonstrated that expiratory flow limitation is the hallmark of COPD, and its most important consequence is lung hyperinflation [32, 33]. Dynamic hyperinflation also impairs diaphragmatic function, shortening the diaphragm to a suboptimal length, decrease the diaphragmatic curvature, and reduce the area of apposition of the costal diaphragm with the chest wall, which gradually affects the motion of the chest wall and diaphragm, leading to the reduced motion; dyspnoea and respiratory drive are increased as a result [34–36]. Additionally, COPD patients reduce their activities of daily living to prevent dyspnoea, which negatively affects their quality of life. As previously reported, our study observed a similar loss of diaphragmatic displacement, shortened diaphragm, and decreased diaphragmatic curvature in COPD patients [30, 37–39]. Further, we detected statistically significant differences between the grade groups according to the severity of COPD in MRI-assessed diaphragmatic displacement. Correlation analysis demonstrated that the diaphragmatic displacement was positively correlated with airflow limitation and exercise tolerance; it was negatively correlated with COPD's quality of life (CAT scores). These data imply that hyperinflation of the lung not only increases the lung area and alters the chest wall and diaphragmatic motion but also puts the respiratory muscles at a mechanical disadvantage [38, 40, 41].

We also observed that the MRI-assessed diaphragm thickness at the end of maximal breathing and the diaphragm thickening fraction in the COPD group were lower than in healthy controls; Our data showed that the diaphragm thickening fraction was positively correlated with airflow limitation and exercise tolerance; it was negatively correlated with the quality of life in COPD patients. These data imply that the diaphragm morphology becomes short and flat in COPD patients. The progression of the disease causes the shortening of diaphragm fibbers and decreases the resting diaphragm muscle length. Structural changes would cause the diaphragms to flatten and thin [42, 43]. Elsawy reported that DTF was significantly decreased in the COPD group and decreased with the increasing severity of COPD [44]. The result is consistent with the work of Kazuki Okura, who reported that the thickness of the diaphragm at total lung capacity or the end of maximal inspiration and the change ratio of the diaphragm thickness (ΔTdi%) were significantly lower in the COPD group [45], and also in agreement with Smargiassi et al., who reported progressive reductions in diaphragm thickness and thickening as air trapping increased [46]. Moreover, Rittayamai also proved that compared to controls, patients with COPD had poorer diaphragm function as measured by TFdi-max and DE-max COPD patients, and diaphragm dysfunction was positively correlated with the severity of airflow obstruction [47].

It is well known that acute exacerbations were the most significant adverse events of COPD, punctuating the natural history, which is associated with significant mortality as well as health and socioeconomic burden [48, 49]. Acute exacerbations of COPD are characterized clinically by symptoms of worsening dyspnoea, cough, sputum production, and sputum purulence, as well as by worsening airflow obstruction [48]. It was reported that 22%–40% of COPD patients experience at least one moderate or severe exacerbation yearly, while 9%–16% experience more than one [50, 51]. At the population level, approximately 20% of GOLD 2 (moderate airflow limitation) patients may experience frequent exacerbations requiring treatment with antibiotics and/or systemic corticosteroids [5]. The risk of exacerbations is significantly higher for patients with GOLD 3 (severe) and GOLD 4 (very severe), which corresponds with our results.

Although exacerbations become more frequent and more severe as COPD progresses, the rate at which they occur appears to reflect an independent susceptibility phenotype. The susceptibility and frequent occurrence of COPD are based on multiple factors, such as low FEV1 [52, 53], poor nutritional status [54], and weak muscles [55]. However, the most crucial determinant of frequent exacerbations is a history of exacerbation [5]. The early identification of exacerbations is of utmost importance. Less than one-third of exacerbations are estimated to be reported [21], because most exacerbations are recorded in outpatient and emergency departments during patient admittance. Furthermore, if acute exacerbations with gradual or mild onset occur at home or treatment works well enough to relieve the symptoms, patients will not visit the hospital, and the exacerbation events might not be recorded. Therefore, early detection and appropriate management of AECOPD patients are essential. However, the early detection of acute exacerbations requires considerable human resources, and caregivers face difficulties in personalizing follow-up management. A major step forward is the current availability of mobile health tools and remote monitoring platforms [56]. Which can provide daily symptoms and enable the dynamic collection of data characterizing a patient's physiological and clinical status. Based on this advantage, we also designed a Telehealth-based monitoring system according to Aaron's modeling. We monitored the occurrence of acute exacerbations in out-of-hospital and demonstrated the feasibility of monitoring acute exacerbations with the Telehealth-based monitoring system [26]. We recorded events of exacerbation during the one-year follow-up and explored the relationship between the frequency of acute exacerbation and diaphragm function.

The diaphragm muscle is essential in respiration, and it is vital to understand lung exercise physiology and the mechanics of COPD [57]. The previous study focused on DD in the acute exacerbation of COPD. It was reported that patients with AECOPD have a higher rate of diaphragmatic dysfunction (DD) than healthy controls, which has been ascribed to hyperinflation-induced diaphragm shortening, placing the diaphragm and respiratory muscle at a mechanical disadvantage, which ultimately causes diaphragmatic weakness and a marked increase in the rate of non-invasive ventilation (NIV) [17]. A further study showed that DD has high specificity and sensitivity in predicting NIV failure in AECOPD patients and may help identify patients who need NIV therapy and be helpful in predicting the duration of hospitalization [58]. A recent study explored the value of diaphragm markers for distinguishing AECOPD from stable COPD [18], which is a step forward in the role of the diaphragm between AECOPD.

The diaphragm, which is the main inspiratory muscle, generates a craniocaudal movement of its dome during contraction [59]. The two striking features in COPD, air trapping and lung hyperinflation, impair the function of the diaphragm, shortening its operating length and changing the mechanical linkage between its various parts, thereby placing it at a mechanical disadvantage [60]. Diaphragmatic dysfunction affects the disease's severity and is related to high mortality, poor treatment effects, and prolonged hospital stay in patients with AECOPD, providing a potential target for therapeutic intervention [61]. The main treatment approaches for COPD are pharmacological therapy and pulmonary rehabilitation. Early pulmonary rehabilitation could improve functional exercise capacity, and health-related quality of life has been demonstrated across all grades of COPD severity [62]. In this study, we demonstrate that the DD could predict AECOPD. If the patients' DD parameters were lower than the optimal cut-off value, we should consider who had a high risk of AECOPD and give target diaphragm rehabilitation. Early targeted diaphragm rehabilitation therapy for patients with a high risk of acute exacerbation may help reduce the number of acute exacerbations and hospitalization.

This study had several limitations. First, this study was conducted at a single centre with a relatively small number of participants; therefore, comparative studies involving a larger number of patients are necessary. Second, in our study, the inclusion criteria required patients to have been exacerbation-free for 12 weeks, considering patients MRI examination cooperation. For COPD patients with poor general conditions and lung function, it is difficult to tolerate magnetic resonance examination. However, we may miss some patients with particularly high exacerbation rates, most of them are being in poor lung function. Third, most of the COPD patients in our study are male. The cause of the male-predominant in the cohort may be related to the epidemiology of COPD. According to previously study, the function of the diaphragm differs between men and women. In normoxia, the magnitude of diaphragm fatigue was equivalent in men and women. Conversely, greater fatigue was expressed in female diaphragm compared to males during equivalent cumulative work in hypoxia [63]. Archiza et al. study also showed the greater vulnerability of the female diaphragm to fatigue during hypoxia [64]. Unfortunately, our male-predominant cohort, may not be generalizable to all COPD patients. It required further study. Finally, our study focused on the relationship between diaphragmatic function parameters and the acute exacerbation of COPD. Diaphragmatic function parameters and acute exacerbations were collected primarily during data acquisition, while the relationship between tidal volumes (VT), inspiratory capacity (IC) and diaphragmatic function was ignored. As for SNIP, our hospital cannot measure it at present. We were unable to analyze the correlation between these measures and MRI indices. The future study will investigate the relationship between IC, VT, and diaphragm function.

## Conclusion

Diaphragmatic dysfunction in patients with COPD is characterised by decreased diaphragmatic displacement, chest wall motion, diaphragmatic dome factor, change of lung area and diaphragm thickening fraction, and increased lung hyperinflation. Moreover, it positively correlated with COPD severity. Additionally, the results of multivariate analysis in this study revealed that the chest wall motion and the diaphragm thickening fraction might predict the frequency of acute exacerbations of COPD.

## Supplementary Information

**Additional file 1**. COPD patients clinical and diaphragm function parameters

**Additional file 2**. Correlation between diaphragm function and clinical parameters in the COPD group

**Additional file 3**. Receiver operating characteristic curves of diaphragm function indices predicting the frequency of AECOPD

## Data Availability

The datasets used and/or analysed during the current study are available from the corresponding author on reasonable request.

## Abbreviations

COPD: Chronic obstructive pulmonary disease
AECOPD: Acute exacerbation of COPD
GOLD: Global initiative for chronic obstructive lung disease
ROC: Receiver operating characteristics curve
DD: Diaphragmatic dysfunction
BMI: Body mass index
CAT: Chronic obstructive pulmonary disease assessment test
6MWT: 6-Min walk test
FEV1: Forced expiratory volume at 1 s
FVC: Forced vital capacity
AUC: Area under the curve
NIV: Non-invasive ventilation
mMRC: Modified Medical Research Council
OR: Odds ratio
CI: Confidence interval
