# Supplementary material for: MRI-assessed diaphragmatic function can predict frequent acute exacerbation of COPD: a prospective observational study based on telehealth-based monitoring system
Source: BMC Pulm Med. 2022 Nov 23;22:438. doi: 10.1186/s12890-022-02254-x (PMC9685983; doi:10.1186/s12890-022-02254-x)
Supplement: Supplementary file 1 — Additional file 1. COPD patients clinical and diaphragm function parameters [file 12890_2022_2254_MOESM1_ESM.pdf]

Additional file 1. COPD patients clinical and diaphragm function parameters

Patient number	GOLD grades	AE	Diaphragmatic dome factor （Insp）	Diaphragmatic dome factor （Exp）	Chest wall motion (cm)	Change of lung area （cm²）	Diaphragmatic displacement （cm）	Diaphragm thickening fraction (%)
1	4	4	1.13	1.10	0.58	42.70	2.74	14.49
2	4	5	1.16	1.37	2.32	132.10	3.81	13.59
3	4	8	1.10	1.22	0.63	67.40	1.38	11.30
4	4	2	1.06	0.98	0.31	96.40	4.86	29.06
5	4	5	1.18	1.35	1.78	153.20	2.82	16.98
6	4	4	1.08	1.12	1.42	92.60	3.82	20.40
7	4	7	1.14	1.13	0.27	87.70	1.82	6.46
8	4	6	1.08	1.12	0.91	63.00	3.03	17.99
9	4	5	1.08	1.18	1.03	40.50	2.88	15.23
10	4	3	1.10	1.06	0.86	49.00	1.65	16.64
11	4	3	1.08	1.12	0.24	19.50	1.82	18.56
12	4	6	1.08	1.18	1.68	84.60	2.22	16.29
13	4	5	1.08	1.13	1.28	101.80	2.52	16.88
14	3	4	1.12	1.23	3.15	154.40	2.59	10.86
15	3	3	1.09	1.23	1.43	116.30	2.86	10.17
16	3	1	1.12	1.23	2.80	99.80	5.82	22.40
17	3	1	1.13	1.32	1.73	121.00	2.13	17.91
18	3	5	1.11	1.23	1.56	69.20	2.40	18.66
19	3	4	1.10	1.24	1.54	67.20	1.38	22.76
20	3	1	1.08	1.22	2.24	83.10	3.70	42.92
21	3	3	1.15	1.28	1.89	106.80	2.74	20.45
22	3	5	1.18	1.25	1.20	119.50	2.02	17.20
23	3	0	1.10	1.21	1.23	58.70	4.81	50.12
24	3	1	1.18	1.30	3.50	146.90	5.14	44.84
25	3	5	1.12	1.22	1.27	81.50	2.95	0.85
26	3	4	1.14	1.17	2.47	88.70	2.45	11.51
27	3	3	1.45	2.00	1.21	69.50	3.82	13.64
28	3	1	1.32	1.38	2.56	95.30	3.88	8.95
29	3	1	1.08	1.12	1.85	51.70	1.22	30.52
30	3	1	1.08	1.22	1.87	120.40	4.59	44.86
31	3	1	1.10	1.19	1.98	97.00	3.33	17.04
32	2	1	1.54	1.22	1.28	79.10	4.36	51.28
33	2	2	1.12	1.23	1.67	54.70	3.37	29.11
34	2	2	1.23	1.29	1.12	109.80	6.54	28.00
35	2	1	1.19	1.25	4.15	137.20	3.12	22.22
36	2	1	1.14	1.17	2.32	84.40	2.67	41.40
37	2	2	1.15	1.20	1.34	136.20	7.63	44.98
38	2	0	1.09	1.18	1.76	122.00	5.33	36.73

39	2	2	1.12	1.21	1.51	128.60	3.59	22.91
40	2	1	1.10	1.26	1.34	138.00	6.25	33.33
41	2	2	1.08	1.26	1.52	153.40	4.17	43.91
42	2	1	1.14	1.16	0.82	105.90	6.93	50.60
43	2	2	1.20	1.26	2.13	93.30	2.19	19.05
44	2	1	1.14	1.27	1.28	171.40	8.53	46.79
45	2	2	1.10	1.24	0.99	118.70	2.82	28.01
46	2	0	1.16	1.26	1.95	117.30	5.35	43.46
47	2	0	1.09	1.23	3.68	148.10	7.73	68.40
48	2	1	1.29	1.33	2.77	140.30	4.50	30.54
49	2	1	1.12	1.24	0.97	114.80	6.05	74.22
50	2	2	1.13	1.30	2.40	136.60	3.91	16.48
51	1	1	1.20	1.45	4.78	198.50	4.28	29.90
52	1	0	1.32	1.40	2.82	157.10	6.87	68.88
53	1	0	1.27	1.24	2.74	179.40	8.13	36.46
54	1	2	1.19	1.18	5.03	164.70	3.21	27.84
55	1	1	1.13	1.25	2.36	173.00	6.46	61.88
56	1	0	1.10	1.23	2.99	171.10	9.80	35.70
57	1	1	1.15	1.21	1.75	130.20	6.01	38.81
58	1	0	1.27	1.31	4.61	179.00	7.21	44.77
59	1	0	1.16	1.22	1.56	127.30	5.07	20.31

Abbreviations: AE， acute exacerbation; COPD, chronic obstructive pulmonary disease; GOLD, Global Initiative for Chronic Obstructive Lung Disease.
