# Supplementary material for: MRI-assessed diaphragmatic function can predict frequent acute exacerbation of COPD: a prospective observational study based on telehealth-based monitoring system
Source: BMC Pulm Med. 2022 Nov 23;22:438. doi: 10.1186/s12890-022-02254-x (PMC9685983; doi:10.1186/s12890-022-02254-x)
Supplement: Supplementary file 2 — Additional file 2. Correlation between diaphragm function and clinical parameters in the COPD group [file 12890_2022_2254_MOESM2_ESM.pdf]

Additional file 2. Correlation between diaphragm function and clinical parameters in the COPD group

Variable		diaphragmatic dome factor (Insp)	chest wall motion（cm）	change of lung area（cm^2）	diaphragmatic displacement (cm)	diaphragm thickening fraction
Age (years)	*r*	-0.1	-0.44**	-0.18	0.25	-0.39**
	P	0.462	<0.001	0.171	0.056	0.012
BM（kg/cm^2）	*r*	0.32*	0.32*	0.41**	0.47**	0.39**
	P	0.015	0.012	0.001	<0.001	0.002
Frequency of AE	*r*	-0.23	-0.43**	-0.43**	-0.64**	-0.66**
	P	0.078	0.001	0.001	<0.001	<0.001
CAT scores	*r*	-0.22	-0.32*	-0.38**	-0.41**	-0.39**
	P	0.091	0.012	0.003	0.001	0.002
6MWT(m)	*r*	0.18	0.42**	0.56**	0.56**	0.50**
	P	0.175	0.001	<0.001	<0.001	<0.001
FEV1(L)	*r*	0.290*	0.55**	0.70**	0.58**	0.52**
	P	0.027	<0.001	<0.001	<0.001	<0.001
FEV1/%pred	*r*	0.31*	0.60**	0.75**	0.64**	0.50**
	P	0.016	<0.001	<0.001	<0.001	<0.001
FVC(L)	*r*	0.26	0.52**	0.76**	0.57**	0.47**
	P	0.05	<0.001	<0.001	<0.001	<0.001
FVC/%pred	*r*	0.18	0.52**	0.73**	0.56**	0.44**
	P	0.175	<0.001	<0.001	<0.001	0.001
FEV1/FVC	*r*	0.30*	0.37**	0.31*	0.37**	0.36**
	P	0.02	0.004	0.015	0.003	0.005

$^{*}P<0.05$，$^{**}P<0.01$

Abbreviations: AE, acute exacerbation；BMI, body mass index; COPD, chronic obstructive pulmonary disease; CAT, chronic obstructive pulmonary disease assessment test; 6MWT, 6-minute walk test; FEV1, forced expiratory volume at 1 s; FVC, forced vital capacity.
