# Supplementary material for: MRI-assessed diaphragmatic function can predict frequent acute exacerbation of COPD: a prospective observational study based on telehealth-based monitoring system
Source: BMC Pulm Med. 2022 Nov 23;22:438. doi: 10.1186/s12890-022-02254-x (PMC9685983; doi:10.1186/s12890-022-02254-x)
Supplement: Supplementary file 3 — Additional file 3. Receiver operating characteristic curves of diaphragm function indices predicting the frequency of AECOPD [file 12890_2022_2254_MOESM3_ESM.pdf]

Additional file 3. Receiver operating characteristic curves of diaphragm function indices predicting the frequency of AECOPD

Variables	Cut-Off value	AUC	95%CI	*P*	Sensitivity（%）	Specificity（%）
chest wall motion(cm)	1.705	0.756	0.631 - 0.881	0.001	75.00%	74.20%
change of lung area (cm^2)	119.95	0.711	0.580-0.842	0.005	60.70%	74.20%
diaphragmatic displacement (cm)	4.225	0.833	0.720-0.946	<0.001	75.00%	90.30%
diaphragm thickening fraction	29.51%	0.869	0.772-0.965	<0.001	78.60%	93.50%

Abbreviations: AECOPD, acute exacerbation of chronic obstructive pulmonary disease; AUC, area under the curve; CI: confidence interval
